# Wheat Germination Is Dependent on Plant Target of Rapamycin Signaling

**DOI:** 10.3389/fcell.2020.606685

**Published:** 2020-11-23

**Authors:** Bauyrzhan Smailov, Sanzhar Alybayev, Izat Smekenov, Aibek Mursalimov, Murat Saparbaev, Dos Sarbassov, Amangeldy Bissenbaev

**Affiliations:** ^1^Department of Molecular Biology and Genetics, Faculty of Biology and Biotechnology, Al-Farabi Kazakh National University, Almaty, Kazakhstan; ^2^Scientific Research Institute of Biology and Biotechnology Problems, Al-Farabi Kazakh National University, Almaty, Kazakhstan; ^3^Groupe «Mechanisms of DNA Repair and Carcinogenesis», Equipe Labellisée LIGUE 2016, CNRS UMR 9019, Université Paris-Sud, Gustave Roussy Cancer Campus, Villejuif, France; ^4^Department of Biology, Nazarbayev University, Nur-Sultan, Kazakhstan

**Keywords:** target of rapamycin pathway, gibberellic acid, abscisic acid, α-amylase, ribosomal protein S6 kinase 1, rapamycin, torin1, wheat seed

## Abstract

Germination is a process of seed sprouting that facilitates embryo growth. The breakdown of reserved starch in the endosperm into simple sugars is essential for seed germination and subsequent seedling growth. At the early stage of germination, gibberellic acid (GA) activates transcription factor GAMYB to promote *de novo* synthesis of isoforms of α-amylase in the aleurone layer and scutellar epithelium of the embryo. Here, we demonstrate that wheat germination is regulated by plant target of rapamycin (TOR) signaling. TOR is a central component of the essential-nutrient–dependent pathway controlling cell growth in all eukaryotes. It is known that rapamycin, a highly specific allosteric inhibitor of TOR, is effective in yeast and animal cells but ineffective in most of higher plants likely owing to structural differences in ubiquitous rapamycin receptor FKBP12. The action of rapamycin on wheat growth has not been studied. Our data show that rapamycin inhibits germination of wheat seeds and of their isolated embryos in a dose-dependent manner. The involvement of *Triticum aestivum* TOR (TaTOR) in wheat germination was consistent with the suppression of wheat embryo growth by specific inhibitors of the TOR kinase: pp242 or torin1. Rapamycin or torin1 interfered with GA function in germination because of a potent inhibitory effect on α-amylase and *GAMYB* gene expression. The TOR inhibitors selectively targeted the GA-dependent gene expression, whereas expression of the abscisic acid-dependent *ABI5* gene was not affected by either rapamycin or torin1. To determine whether the TaTOR kinase activation takes place during wheat germination, we examined phosphorylation of a ribosomal protein, *T. aestivum* S6 kinase 1 (TaS6K1; a substrate of TOR). The phosphorylation of serine 467 (S467) in a hydrophobic motif on TaS6K1 was induced in a process of germination triggered by GA. Moreover, the germination-induced phosphorylation of TaS6K1 on S467 was dependent on TaTOR and was inhibited by rapamycin or torin1. Besides, a gibberellin biosynthesis inhibitor (paclobutrazol; PBZ) blocked not only α-amylase gene expression but also TaS6K1 phosphorylation in wheat embryos. Thus, a hormonal action of GA turns on the synthesis of α-amylase in wheat germination via activation of the TaTOR–S6K1 signaling pathway.

## Highlights

- Rapamycin or ATP-competitive TOR inhibitors effectively inhibit germination of wheat whole seeds and isolated embryos dose-dependently.
- TOR-S6K1 signaling is important for regulation of GA-induced mobilization of starch and for seedling growth.

## Introduction

The breakdown of reserved starch in the endosperm into simple sugars is an essential step for seed germination and subsequent seedling growth. At the early stage of germination, gibberellic acid (GA) activates the myb-like transcription factor (GAMYB) that promotes *de novo* synthesis of α-amylase in the aleurone layer and embryo. α-Amylase expression in the embryo is localized to the scutellar epithelium (Kaneko et al., 2002). Furthermore, GA stimulates *de novo* synthesis of proteases and peptidases and ∼50% of total protein synthesis during germination (Bak-Jensen et al., 2007). Among hydrolases, abundant α-amylases play a central role in the metabolizing of starch that determines the rate of germination and seedling growth. Abscisic acid (ABA) represses most effects of GA including α-amylase expression in aleurone and embryonic tissues (Gomez-Cadenas et al., 2001). GA induces α-amylase expression in rice (Gomi et al., 2004) and barley (Fu et al., 2002) aleurone cells through proteasome-dependent degradation of DELLA proteins (SLR1, slender rice-1 in rice and SLN, slender 1 in barley) mediated by receptor GID1. Furthermore, it has been shown that prior to the establishment of the GID1–DELLA and GA perception system, the effect of GA on α-amylase mRNA expression is preceded by increases in cytosolic free Ca^2+^ concentration and changes in cytosolic pH and in the concentrations of calmodulin and cyclic GMP (Bethke et al., 1997). Experiments with an agonist and inhibitor of heterotrimeric G proteins point to their involvement in the oat aleurone response to GA (Jones et al., 1998). Recently, it was reported that reactive oxygen species perform a key function in the regulation of α-amylase production in barley aleurone cells (Aoki et al., 2014). Nonetheless, how GA regulates α-amylase synthesis in wheat germination remains poorly characterized.

Target of rapamycin (TOR) is a central regulator of cell proliferation and growth in eukaryotic cells. TOR integrates signals from multitude inputs such as growth factors, stress, nutrients, and energy to regulate protein synthesis, energy metabolism, cell cycle progression, and autophagy (Saxton and Sabatini, 2017). In animals and yeast, TOR is present in at least two structurally and functionally distinct multiprotein complexes: TORC1 and TORC2 (Loewith and Hall, 2011; Laplante and Sabatini, 2012). TORC2 does not exist in plants (Shi et al., 2018). The core components of mammalian TORC1 are mammalian TOR (mTOR), RAPTOR (regulatory-associated protein of mTOR), and mLST8 (mammalian lethal with SEC13 protein 8). The stability of interactions among mTOR, RAPTOR, and mLST8 is sensitive to nutrient and energy levels (Saxton and Sabatini, 2017). The best-characterized effector of TORC1 is a translational regulator (a 40S ribosomal protein) called S6 kinase 1 (S6K1) (Pearce et al., 2010). It regulates translation initiation by phosphorylating the S6 protein of the 40S ribosomal subunit and by stimulating eIF-4A helicase activity (Holz et al., 2005). Some studies indicate that animal S6K participates in regulation of fundamental cellular processes, including transcription, translation, protein and lipid synthesis, cell growth, cell size determination, and cell metabolism (Saxton and Sabatini, 2017). In contrast to animal S6Ks, there are a few studies characterizing plant S6Ks despite the importance of their function. A key role of S6K in chromosome stability and regulation of cell proliferation was shown in *Arabidopsis* (Henriques et al., 2010; Schepetilnikov et al., 2011). In addition, *Arabidopsis* S6K1 activation by TOR is important for reinitiating the translation of an mRNA that contains upstream open reading frames in 5′-untranslated regions (Schepetilnikov et al., 2013). The TOR–RAPTOR2–S6K1 complex is critical for the modulation of thylakoid membrane lipid biosynthesis and homeostasis in rice (Sun et al., 2016). Elucidation of TOR signaling has been advanced by studies on the mechanism of action of rapamycin. Rapamycin is a bacterially produced macrolide that inhibits TOR and has a variety of clinical applications such as antifungal, immunosuppressant, and anticancer treatments. In mammalian systems, most of the known mTOR substrates were discovered and validated using rapamycin as a pharmacological probe (Thoreen et al., 2009). Rapamycin forms a complex with its intracellular receptor FK506-binding protein 12 kDa (FKBP12), and the complex binds to a specific region of TOR (defined as the FKBP12–rapamycin binding domain; FRB) and thereby inhibits the kinase activity of TOR. Although rapamycin is a highly specific allosteric inhibitor of mTOR, it is only a partial inhibitor of this enzyme. Rapamycin targets mTORC1 but not mTORC2 (Sarbassov et al., 2004, 2006). Torin1 is a synthetic mTOR inhibitor that blocks ATP binding to mTOR and thus inactivates both mTORC1 and mTORC2. Torin1 and rapamycin each inhibits overall protein synthesis, induces autophagosome formation, and thus mimics the effects of starvation (Thoreen et al., 2009; Zhao et al., 2015). Despite the connections of mTORC1 to translational machinery, the effects of rapamycin on mammalian cell growth and proliferation are much less dramatic than the effects on yeast growth and proliferation and are highly dependent on the cell type (Barbet et al., 1996; Thoreen et al., 2009).

Plant TOR may be poorly sensitive to rapamycin as a result of mutations in the *Arabidopsis thaliana* FKBP12 protein that prevent the assembly of inhibitory complex TOR–rapamycin–FKBP12 (Ren et al., 2011, 2012). On the other hand, some studies have shown that tomato (Xiong et al., 2016) and *A. thaliana* seedlings are sensitive to micromolar doses of rapamycin (Xiong and Sheen, 2012; Xiong et al., 2013). The phosphorylation of S6K1 threonine 449 (T449) was shown to be inhibited when 100 nM torin1 was added for a 30-min incubation to transfected protoplasts transiently expressing *A. thaliana* S6K1 (AtS6K1) tagged with FLAG (Xiong et al., 2013). In a yeast reconstitution model, it was shown that rice S6Ks restore ribosomal-protein S6’s phosphorylation in a rapamycin-sensitive manner in yeast cells lacking Ypk3, an ortholog of mammalian S6Ks (Yaguchi and Kozaki, 2018). Furthermore, transcriptome analysis of rice cells revealed that rapamycin treatment significantly suppress expression of 120 genes encoding histones, chromatin modulators, ribosomal proteins, and protein synthesis machineries (De Vleesschauwer et al., 2018). These observations suggest that plant TOR plays an important part in the regulation of transcriptional and translational processes just as in other eukaryotes.

A loss-of-function mutation of *Raptor1b* leads to a substantial delay in *Arabidopsis* seed germination; delayed germination and seedling growth in Raptor1b-null seeds are partially restored by an exogenous supply of GA (Salem et al., 2018). In the present study, we examined the effects of mTOR inhibitors (rapamycin or torin1) on the germination of wheat seeds and on α-amylase gene expression in the wheat embryos.

## Materials and Methods

### Wheat Growth Conditions

The wheat (*Triticum aestivum*, variety Kazakhstanskaya 19) seeds were sterilized in 2% (v/v) NaOCl for 20 min and washed twice with sterile water, once with 0.01 M HCl, and thoroughly with sterile distilled water. The surface-sterilized seeds were germinated under long-daylight conditions at room temperature on filter paper soaked with sterile water supplemented with 1 μM GA and with or without various concentrations of rapamycin or torin1 (0.5, 1.0, 5.0, or 10.0 μM). Primary roots, shoots, and seedlings were photographed, and shoot length and fresh weight of seedlings were measured at 4 days after germination (DAG). In addition, the embryos isolated from 1-DAG seeds were transferred to the 0.5× MS medium in Petri dishes with 1 μM GA or 10 μM ABA and different inhibitors (rapamycin, torin1, or pp242) for 4-day incubation, unless specified otherwise.

### Wheat Embryo Incubation Conditions

Embryos were dissected from 1-DAG seeds by means of a scalpel. Only intact embryos with no starch or aleurone tissue adhering to the scutellar tissue were studied. Twenty embryos were incubated in each well of six-well plastic plates in 2 ml of 10 mM CaCl_2_ with 2.5 μg/ml of chloramphenicol. Embryos were incubated up to 24 h at room temperature in the dark with constant shaking at 125 rpm. GA and ABA were purchased from Sigma-Aldrich (Germany). GA was applied at 1 μM and ABA at 10 μM. Assays were carried out in the presence or absence of various concentrations of rapamycin (LC Laboratories, Woburn, MA, United States) or torin1 (Cayman Chemical Company, Ann Arbor, MI, United States) or 100 μM PBZ (Sigma-Aldrich, Germany). Rapamycin and torin1 were dissolved in DMSO and stored at −20°C according to the manufacturer’s instructions. The rapamycin solution was preheated to 37°C prior to its application.

### RNA Isolation and cDNA Synthesis

The *T. aestivum* cDNA encoding putative ribosomal protein S6 kinases (*tas6k1*) was identified by its homology to rice S6K1 (GenBank accession No. AK451448.1). TaS6K1 cDNA was prepared by reverse-transcription polymerase chain reaction (RT-PCR) with gene-specific primers (Table 1). Total RNA was isolated from young leaves of *T. aestivum* variety Kazakhstanskaya 19 by the TRIzol method. The yield and purity of RNA were determined spectrophotometrically, and the quality of RNA was evaluated by electrophoresis in a 1% formaldehyde agarose gel. First-strand cDNA was synthesized by reverse transcription from 5 μg of total RNA as a template under the following conditions: 200 U of RevertAid M-MuLV reverse transcriptase (Thermo Scientific, Lithuania), 0.5 μg of the oligo-dT_18_ primer, and 1 μM dNTPs in a final volume of 20 μl. An aliquot of the first-strand cDNA served as a template in the PCR for the synthesis of second-strand cDNA, and subsequent amplification of double-stranded cDNA was performed with designed gene-specific primers. The amplicons were separated by electrophoresis in a 1% agarose gel, and the product of expected size was extracted from the gel using the Silica Bead DNA Gel Extraction Kit (Thermo Scientific, Lithuania). The fragment was cloned into the pBluescript II SK (+) vector at *Eco*RI and *Bam*HI restriction sites by means of the Rapid DNA Ligation Kit (Thermo Scientific, Lithuania), and the ligation product was transfected into competent *Escherichia coli* DH5α cells. Colonies of *E. coli* carrying the plasmid with an insert were screened out by complementation of the *lacZ* gene, and the plasmid DNAs were isolated with the GeneJET Plasmid Miniprep Kit (Thermo Scientific, Lithuania). The presence of the insert in the isolated plasmids was confirmed by PCR with gene-specific primers, and its sequence was confirmed by sequencing in both directions with forward and reverse M13 primers.

**TABLE 1:** Primers employed in this study.

Primers	DNA sequence (5′-3′)	Amplicon length (bp)
TaS6K_Fw	TATCGAATTCACATGGTTTCCTCTGAG	1,475
TaS6K_Rev	ATCACCCGGGGGATCCTTAGCCTAGAG	
Amy1–3a_Fw	ATGTGGCCCTTCCCTTCCGA	409
Amy1–3a_rev	TGGATGTCCCTCATCCTCACTTTTACA	
TaGamyB_Fw	GGTGGACTACGTGAAGAAGC	369
TaGamyB_Rev	GATTTTCGCCGCAGTTGAAATCGC	
TaABI5 _Fw	TGACGCTGGAGCAGTTTCTT	332
TaABI5_Rev	TCGCCCATGCAGTTCATCAT	
α-Tubilin_Fw	ATCTCCAACTCCACCAGTGTCG	219
α-Tubilin_Rev	TCATCGCCCTCATCACCGTC	
RHT_SmaI_Fw	ATTATCCCGGGATGAAGCGGGAGTACCA	1,895
RHT_HindIII_Rev	TATTCAAGCTTTCACGGCCCGGCCAGG	

Primers used for RT-PCR (PCR) are listed in Table 1. RT-PCR amplification of a 409 bp fragment of α-amylase cDNA was performed using Amy1-3a_Fw and Amy1-3a_Rev as a forward and reverse primer, respectively. RT-PCR amplification of the 369-bp fragment representing cDNA of *GAMYB* was performed with TaGamyB_Fw and TaGamyB_Rev as a forward and reverse primer, respectively. RT-PCR amplification of *T. aestivum* ABA response element-binding factor (*TaABI5*) cDNA (332 bp) was performed with TaABI5_Fw and TaABI5_Rev as a forward and reverse primer, respectively. All amplifications were carried out with Taq DNA polymerase (Promega, Madison, WI 53711, United States). The resulting PCR products were resolved by electrophoresis in a 2% agarose gel and visualized by ethidium bromide staining.

### Expression and Purification of 6xHis-Tagged TaS6K1

The cDNA encoding TaS6K1 was subcloned into the pET28c vector at *Eco*RI and *Not*I sites resulting in expression plasmid pET28c-TaS6K1, which carries an N-terminal 6xHis-tag sequence. The wheat recombinant TaS6K1 (rTaS6K1) protein was expressed in (and purified from) the *E. coli* Rosetta (DE3) strain. Briefly, *E. coli* cells were electroporated with pET28c-TaS6K1, the resultant kanamycin-resistant transformants were grown to optical density at 600 nm of 0.6 at 37°C, and protein expression was then induced by 0.1 mM isopropyl β-D-1-thiogalactopyranoside (IPTG) overnight at 30°C. Due to high expression in the Rosetta strain, it was possible to purify rTaS6K1 to homogeneity via only two chromatographic steps. All the purification procedures were carried out at 4°C. The bacteria were harvested by centrifugation, and the cell pellets were lysed using a French press at 18,000 psi in a buffer consisting of 20 mM HEPES-KOH (pH 7.6), 50 mM KCl, and a Complete Protease Inhibitor Cocktail (Roche Diagnostics, Switzerland). The lysates were cleared by centrifugation at 40,000 × *g* for 30 min at 4°C, and the buffer in the resulting supernatant was adjusted to 500 mM NaCl and 20 mM imidazole. The protein sample was loaded onto a HiTrap Chelating HP column (GE Healthcare) charged with Ni^2+^. The eluted fractions containing rTaS6K1 were pooled and loaded onto a 1-ml HiTrap-Heparin column (GE Healthcare). The bound proteins were eluted in a 50–600 mM KCl gradient. The purified protein samples were stored at −20°C in 50% glycerol. The homogeneity of the protein preparations was verified by SDS-PAGE (Supplementary Figure S1A).

### Preparation of an Anti-TaS6K1 Antibody

The anti-TaS6K1 polyclonal antibody was raised against the full-length 6xHis-tagged rTaS6K1 protein. Approximately 0.5 mg of the purified rTaS6K1 was mixed with an equal volume of Freund’s complete adjuvant (F5881, Sigma-Aldrich, Canada) and injected at five spots on the back of each rabbit. Three additional injections were made at 2-week intervals. 1 week after the last injection, blood was collected, and ammonium sulfate was added to 3 ml of the obtained rabbit antiserum to achieve 50% saturation. The precipitate was collected by centrifugation, and the pellet was dissolved in purified water and dialyzed against 10 mM potassium phosphate buffer (pH 7.0). The obtained immunoglobulin fraction was loaded on a column with protein A agarose beads equilibrated with the abovementioned buffer. After a wash with the same buffer, antibodies were eluted with 100 mM glycine buffer (pH 3.0). The IgG-containing fractions were pooled, and pH was adjusted to 7.0 with 1.0 M Tris base. The resulting suspension was kept at 4°C. This antibody was evaluated in an optimized Western blot assay, and the obtained signal was very strong even when the antibody was diluted 1:20,000.

### Antiserum Titer Determination by an ELISA

The titers of antisera were determined by an indirect ELISA. Each well of a 96-well ELISA plate (Corning Inc., United States) was coated with 1 μg of rTaS6K1 dissolved in 100 μl of 50 mM carbonate-bicarbonate buffer (pH 9.6) via overnight incubation at 4°C. After three washes with phosphate-buffered saline (PBS) Tween buffer (PBST; 0.05% of Tween 20 in PBS, pH 7.4), the wells were blocked with 100 μl of 3% BSA in PBST for 1 h at 37°C and then washed again twice with PBST. After blocking, 100 μl of serially diluted anti-TaS6K1 serum (1:1,000 to 1:128,000) was added into the antigen-coated wells. The plate was covered with an adhesive plastic and incubated for 2 h at room temperature and then washed four times with PBST. At the next step, a 1:30,000-diluted alkaline phosphatase-conjugated goat anti-rabbit IgG antibody (Sigma, Canada) was added at 100 μl/well and incubated for 1 h at 37°C. After a wash, 100 μl of a freshly prepared p-nitrophenyl phosphate (substrate) solution was added into each well, and the plate was incubated at room temperature in a dark place. Finally, absorbance was measured at 405 nm (A_405_) on a Multiskan FC plate reader (Thermo Scientific, Waltham, MA, United States). The antibody titer is defined as the highest dilution of antiserum at which the ratio of A_405_ (A_405_ of post-immunization serum/A_405_ of preimmunization serum) is >2:1. All samples were tested in triplicate, with each plate containing control wells with positive serum samples and control wells with negative reference serum.

### Plant Protein Extraction and Western Blotting

Wheat embryos were ground in liquid nitrogen and then resuspended in lysis buffer A consisting of 50 mM Tris–HCl, 50 mM sodium β-glycerophosphate (pH 7.6), 25 mM EDTA, 25 mM EGTA, 50 mM NaF, 5 mM Na_3_VO_4_, 10% of glycerol, 1% of Triton X-100, 1 mM phenylmethylsulfonyl fluoride, and EDTA-free protease inhibitors (Roche Applied Science). The cell debris was pelleted, and the protein concentration was determined using the Bradford Protein Assay Kit (Bio-Rad, France). Total protein samples (25 μg) from each extract were fractionated by SDS-PAGE in a 10% gel and then electroblotted onto a polyvinylidene difluoride (PVDF) membrane (Pierce) by means of a Bio-Rad Mini-Trans-blot Cell. After that, the membrane was gently shaken in a blocking solution consisting of 5% milk and 0.1% Tween 20 in 1× TBS [Tris-buffered saline: 50 mM Tris–HCl (pH 7.5) and 20 mM NaCl] for 1 h at room temperature. After removal of the blocking solution, the membrane was incubated in 10 ml of a 20,000-fold dilution of either the anti-α-amylase polyclonal antibody or anti-TaS6K1 polyclonal antibody overnight at 4°C. The membrane was washed five times in 10 ml of wash buffer (1× TBS with 0.1% of Tween 20) for 5 min each time. Next, the membrane was incubated in 10 ml of a secondary antibody (1:30,000 dilution in the blocking solution with 0.1% of Tween 20) for 1 h at room temperature. Then, the membrane was washed five times in 10 ml of wash buffer, for 5 min each time. The working substrate solution was prepared by mixing an equal volume of a peroxide solution and luminal/enhancer solution and was used at 0.1 ml/(cm^2^ of the blot area). The membrane was incubated in the working solution for 2 min in darkness, and Kodak X-Omat was exposed to the film. The polyclonal antibody to wheat α-amylase was kindly provided by Dr. A. Khakimzhanov (Aitkhozhin Institute of Molecular Biology and Biochemistry, Kazakhstan).

For analyses of phosphorylation of TaS6K1 in cell-free extracts, the embryos were dissected from 1-DAG seeds pre-treated with 100 μM PBZ for 20 h and then were incubated with 10 mM CaCl_2_ and 1 μM GA in the absence or presence of 10 μM rapamycin. The wheat embryos were ground in liquid nitrogen and then resuspended in lysis buffer B consisting of 50 mM Tris–HCl (pH 7.6), 150 mM NaCl, an EDTA-free phosphatase inhibitor cocktail, 1 mM phenylmethylsulfonyl fluoride, 10 μg/ml of leupeptin, and 1 μg/ml of aprotinin. Samples of cell lysates containing 10 μg of protein were separated by SDS-PAGE in a 10% gel and transferred to a 0.2-μm PVDF membrane (Pierce). Membranes were blocked with 5% BSA for 2 h in TBS with 0.02% of Tween 20 and probed overnight with an anti-phospho-p70 S6 kinase (T389) antibody (1:1,000; Cell Signaling Technology, Danvers, MA, United States) and the anti-TaS6K1 antibody (1:20,000), followed by 1 h incubation with secondary antibodies coupled to peroxidase (1:10,000 and 1:30,000, respectively).

### Zymogram Analysis of α-Amylase

Non-denaturing polyacrylamide gel electrophoresis (native PAGE) was performed according to the standard method (Laemmli, 1970) to obtain a zymogram of α-amylase. Wheat embryos were ground in liquid nitrogen and then resuspended in 10 mM CaCl_2_. The samples were mixed with 50% saccharose and loaded onto a polyacrylamide gel (4 and 10% polyacrylamide for the stacking and resolving gels, respectively). Electrophoresis was carried out at 100 V and 4°C. After that, the gel was incubated in 10 mM CaCl_2_ for 30 min at room temperature. Then, the gel was incubated in a 1% (w/v) starch solution at 30°C and shaken for 60 min. After the gel was washed with distilled water, it was stained with the Lugol solution (1.3% I_2_ and 3% KI). The bands of α-amylase activity appeared as bright bands on a dark background and were photographed.

### Immunoprecipitation of TaS6K1

Wheat embryos were ground in liquid nitrogen and then resuspended in lysis buffer A. After centrifugation at 13,000 × *g* for 10 min, 4 μg of the anti-TaS6K1 polyclonal antibody was added to the cleared supernatant and incubated with rotation for 6 h. Then, 20 μl of a 50% slurry of protein G-Sepharose was added, and the incubation was continued for 4 h. Captured immunoprecipitates were washed three times with lysis buffer. Samples were resolved by SDS-PAGE, and proteins were transferred to a PVDF membrane and visualized by immunoblotting with either the anti-TaS6K1 polyclonal antibody or phospho-AtS6K antibody (p-T449; Abcam, Cambridge, United Kingdom). A goat anti-rabbit IgG (Abcam, Cambridge, United Kingdom) or mouse anti-rabbit IgG (Conformation Specific; Cell Signaling Technology, Danvers, MA, United States) antibody, respectively, was used to detect the primary antibodies.

### Statistical Analysis

Data were analyzed for statistical significance by the application of complete randomized design with three replicates. The two-tailed *t* test, assuming equal variances, was conducted to determine whether the differences were statistically significant. Data with a value of *P* < 0.05 were deemed significant.

## Results

### Effects of Rapamycin or Another mTOR Kinase Inhibitor on the Growth of Wheat Whole Seeds and of Isolated Wheat Embryos

**FIGURE 1:**
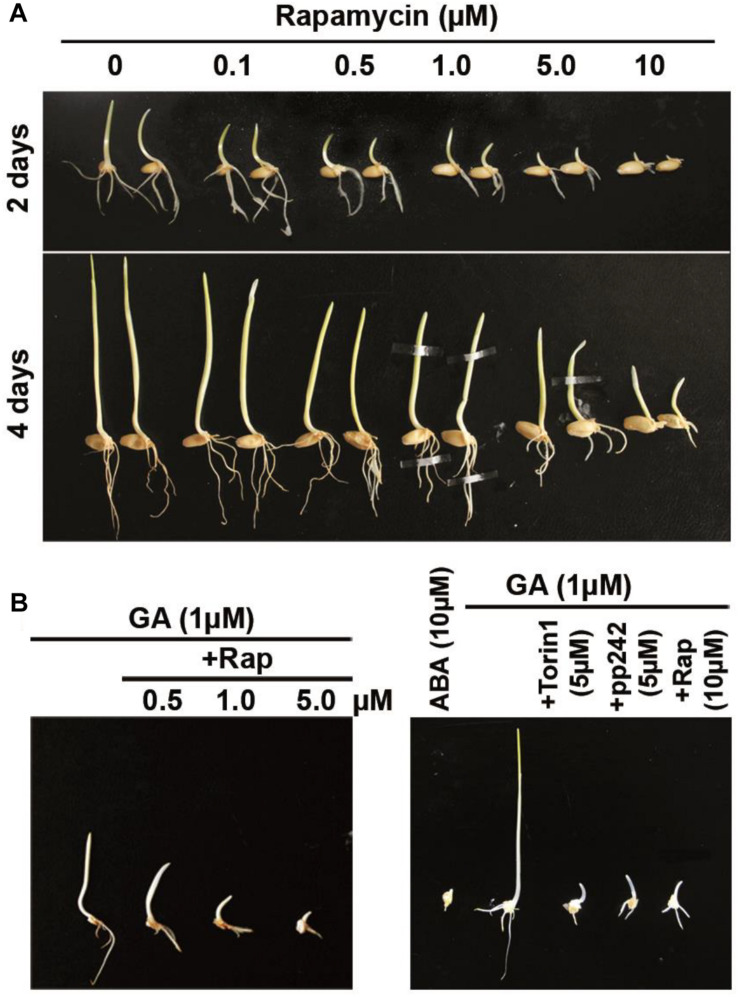
Rapamycin and ATP-competitive target of rapamycin (TOR) inhibitors exert inhibitory effects on the growth of wheat whole seeds and of isolated wheat embryos. **(A)** The phenotype of seedlings after 2 and 4 days of germination in the presence of rapamycin. The surface-sterilized seeds were germinated under long-daylight conditions at room temperature on filter paper soaked with sterile water supplemented with rapamycin at different concentrations for 2 and 4 days. **(B)** Effects of rapamycin and ATP-competitive TOR inhibitors on isolated wheat embryos. The embryos were incubated on a 0.5× MS medium with 10 μM ABA or 1 μM GA alone and in combination with rapamycin, torin1, or pp242 for 4 days.

**FIGURE 2:**
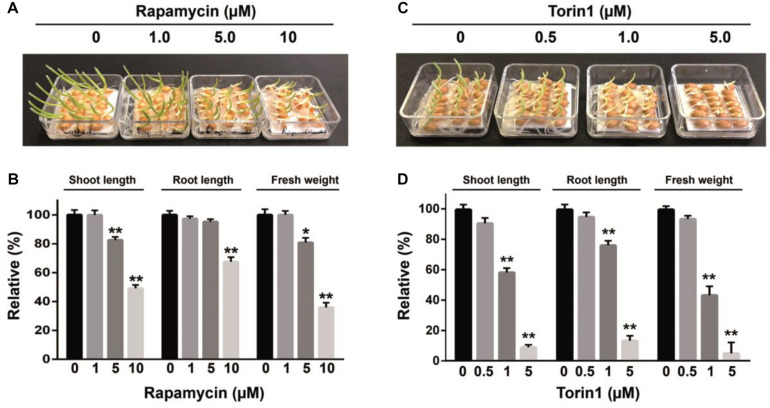
The inhibitory effects of rapamycin and torin1 on growth of wheat whole seeds. The phenotype of seedlings after 4 days of germination in the presence rapamycin **(A)** or torin1 **(C)**; the fresh weight of seedlings and primary root and shoot lengths shown in panels **(A,C)** were measured and presented in panels **(B,D)** accordingly. The surface-sterilized seeds were germinated under long-daylight conditions at room temperature on filter paper soaked with sterile water supplemented with rapamycin or torin1 at different concentrations for 4 days. Values represent mean ± SEM. Asterisks indicate significant differences from the DMSO treatment (0.1%), according to Student’s *t* test (*P < 0.05, **P < 0.01). Each studied drug concentration corresponds to three biological replicates.

In initial experiments, we assessed the effects of rapamycin on the germination capacity of intact wheat seeds. Germination of wheat seeds was tested in the presence of various concentrations of rapamycin. The effects of rapamycin on seed growth were observed after 2 and 4 days (Figure 1A). Rapamycin at 0.1–1.0 μM caused a relatively weak inhibition of the germination of caryopses. Significant retardation of radicle emergence was observed in the presence of 5 or 10 μM rapamycin following 2 days of treatment. Although the rapamycin-treated seeds were able to germinate after 4 days, we observed potent retardation of seedling growth by 10 μM rapamycin leading to a substantial reduction in fresh weight of seedlings and in root and shoot length (*P* < 0.01). Figures 2A,B shows that rapamycin at 10 μM caused a reduction in the length of shoots by 49.17%, length of roots by 67.47%, and fresh weight of seedlings by 64% when compared with the control (DMSO-treated) seeds.

In yeasts and animals, rapamycin has been used extensively to dissect the TOR pathway because of the ability to specifically inhibit TOR activity (Thoreen et al., 2009). Nonetheless, in comparison with yeast or mammalian cells growth inhibition of wheat seeds requires much higher concentrations of rapamycin (Vezina et al., 1975; Kuo et al., 1992). Recently, new mTOR kinase inhibitors were developed that are more effective than rapamycin in cancer therapy (Zhang et al., 2011). The new TOR kinase inhibitors are ATP-competitive compounds targeting the ATP-binding pocket within the mTOR kinase domain (Zhou et al., 2010). The mTOR kinase inhibitors have been successfully applied to inhibit TOR signaling in plants (Montane and Menand, 2013; Dong et al., 2015; Xiong and Sheen, 2015; Xiong et al., 2016). We found that the suppressive effect of torin1 on wheat germination was more potent compared to the effects of rapamycin, namely, the torin1-treated wheat seedlings contained a smaller cotyledon with a significant concentration-dependent reduction in seedling growth. A substantial decrease in shoot (91%) and root length (86.76%) compared to the control was observed after 5 μM torin1 treatment. At the 1 μM concentration, its inhibitory effect decreased to 41.64 and 24%, respectively, and only a weak effect of torin1 was detectable at 0.5 μM indicating only a 9.1 and 5% decrease, respectively. The strong inhibitory impact of torin1 at 5 μM was consistent with a dramatic 95% decrease in fresh weight of seedlings, which was only 56.7% at 1 μM torin1 (Figures 2C,D).

The germination of isolated wheat embryos also indicated sensitivity to the mTOR inhibitors. To investigate the sensitivity of embryo growth to rapamycin, the isolated embryos were transferred to the 0.5× MS medium with 10 μM ABA or 1 μM GA alone and in combination with rapamycin, torin1, or pp242 (Figure 1B). Naturally occurring growth inhibitor ABA was very effective at repressing embryo growth judging by the growth of untreated control embryos. We found that rapamycin at 0.5 μM inhibited embryo growth even after 4 days of incubation, meaning its more potent impact on isolated wheat embryos. The inhibitory influence of rapamycin on embryo growth was more pronounced at its higher concentrations (5 and 10 μM). Besides, the embryo growth was significantly inhibited by mTOR kinase inhibitor torin1 or pp242 (Figure 1B). In the absence of GA, mTOR kinase inhibitors also strongly inhibited the embryo growth (Supplementary Figure S2).

Taken together, these results strongly indicated that the growth of wheat seeds is dependent on *T. aestivum* TOR (TaTOR) signaling.

### Rapamycin and Torin1 Each Inhibits the Expression of GA-Induced α-Amylase and *GAMYB* Genes but Not the ABA-Induced *TaABI5* Gene

**FIGURE 3:**
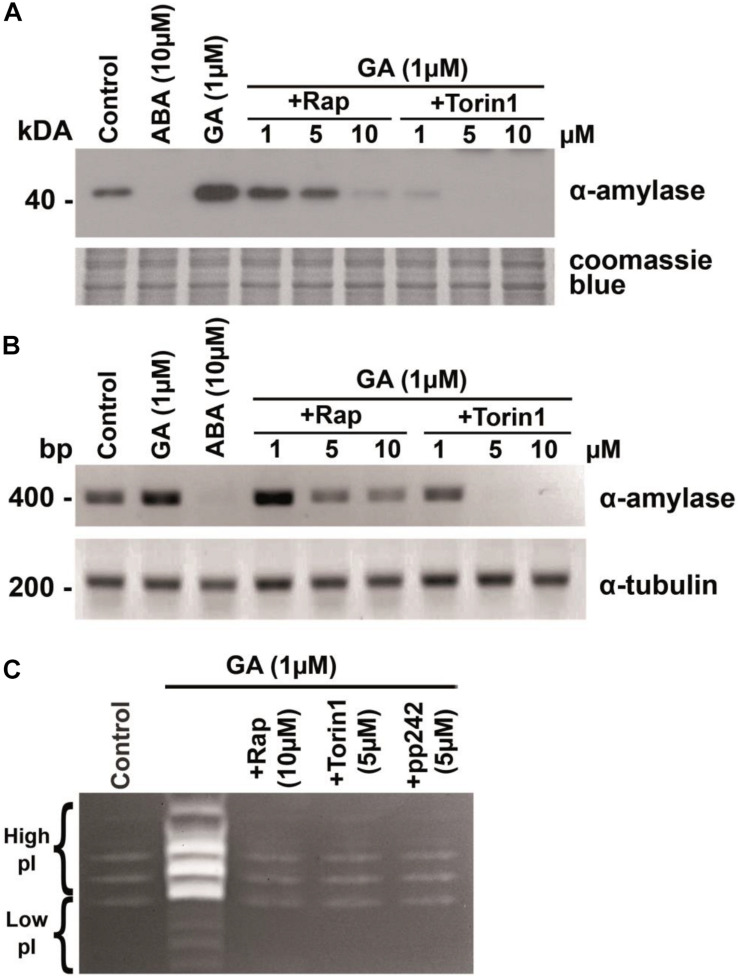
Gibberellic acid (GA)-induced expression of the α-amylase gene is attenuated in the presence of TOR kinase inhibitors. **(A)** Concentration-dependent influence of rapamycin or torin1 on GA-induced production of α-amylase. The wheat embryos dissected from 1-DAG seeds were incubated for 24 h in the presence 10 mM CaCl_2_ (control) with or without 10 μM abscisic acid (ABA), or 1 μM GA, or 1 μM GA with different concentrations of rapamycin or torin1. The embryos were then lysed and subjected to immunoblotting. Rap, rapamycin. **(B)** Concentration-dependent effects of rapamycin and torin1 on GA-induced accumulation of α-amylase mRNA. The wheat embryos were incubated as described in panel **(A)**. A 409-bp fragment of α-amylase cDNA was amplified by RT-PCR with gene-specific primers. α-Tubulin expression served as an internal loading control. **(C)** Detection of α-amylase activity by staining. The wheat embryos were incubated for 24 h in the presence of 1 μM GA and 10 mM CaCl_2_ with or without rapamycin, torin1, or pp242. The embryos were then lysed and subjected to staining for the activity of α-amylases following partition in a polyacrylamide gel electrophoresis (native PAGE).

The analysis of wheat embryo extracts uncovered a significant increase in α-amylase levels in embryos treated with 1 μM GA_3_ for 24 h compared with the extract obtained from the untreated (control) embryos. The addition of 10 μM ABA to the incubation medium abrogated α-amylase production (Figure 3A). A dose-dependent effect of rapamycin on the GA-induced α-amylase production meant that rapamycin was effective at concentrations between 1 and 10 μM. Rapamycin at the 1 or 5 μM concentration had a weak effect on α-amylase production, whereas 10 μM rapamycin completely blocked the GA-induced α-amylase synthesis. We also examined the influence of torin1. As shown in Figure 3A, the incubation of embryos with torin1 prevented the GA-induced α-amylase accumulation in the concentration range from 1 to 10 μM.

The observation that the mTOR inhibitors were sufficient to block GA-induced α-amylase production in wheat embryos suggested that the TOR kinase activity is required for GA signaling. To determine whether TOR kinase activation is required for α-amylase mRNA accumulation, wheat embryos were incubated with 1 μM GA, 10 μM ABA, 1 μM GA + rapamycin (1–10 μM), or 1 μM GA + torin1 (1–10 μM) for 24 h, and relative levels of α-amylase gene expression were examined by RT-PCR. We found that GA induced a high level of α-amylase mRNA accumulation compared with untreated embryos (Figure 3B). The influence of rapamycin and of torin1 on α-amylase mRNA accumulation was concentration dependent. Their dose–response pattern was almost identical to that noted for the α-amylase protein production in wheat embryos, as presented in Figure 3A. The expression profile of the α-tubulin gene was not altered by rapamycin or torin1 treatment. Therefore, the expression data are consistent with the above observations where rapamycin or torin1 inhibited the GA-induced α-amylase production, suggesting that the mTOR inhibitors suppress the induction of α-amylase gene expression and prevent the α-amylase enzyme synthesis (Figure 3A).

The major α-amylases in wheat (*T. aestivum*) are the high- and low-isoelectric point (pI) α-amylases encoded by two multigene families (α-Amy-1 and α-Amy-2 genes) located on chromosomes 6 and 7, respectively. Interestingly, the α-amylase isoenzymes in wheat cultivars differ in their pI but not in molecular weight (∼43 kDa) (Cheng et al., 2014). During wheat seed germination, high-pI α-amylase is produced at a higher concentration than low-pI α-amylase (Amy2) (Ju et al., 2019). In our study, the effect of GA was accompanied by the appearance of strong distinct bands corresponding to high-pI α-amylase on the electropherogram. Rapamycin and ATP-competitive inhibitors of TOR kinase abrogated this effect of GA (Figure 3C).

Because we observed a decrease in α-amylase gene expression and α-amylase activity after the inhibition of TOR, we tested whether the GA-induced *GAMYB* mRNA accumulation is sensitive to rapamycin or torin1. In cereals, GA regulates α-amylase synthesis in the aleurone and scutellar epithelium via induction of transcription factor GAMYB, which binds to a highly conserved GA-responsive element (GARE) in the promoters of α-amylase genes (Gubler et al., 1995; Washio, 2003). To assess the impact of TOR signaling on the GA-dependent GAMYB expression, we measured levels of *GAMYB* and α-amylase mRNAs in wheat embryos incubated with or without GA and an mTOR inhibitor. As depicted in Supplementary Figure S3, the levels of *GAMYB* and α-amylase mRNAs were induced dramatically by GA following 24 h of incubation. ABA caused an opposite effect by reducing the levels of *GAMYB* and α-amylase mRNAs that were associated with the induction of *TaABI5* mRNA expression. Although the rapamycin or torin1 treatment led to a substantial reduction of α-amylase and *GAMYB* mRNAs levels in GA-treated wheat embryos, neither mTOR inhibitor abolished ABA-dependent expression of TaABI5. This finding showed that the mTOR inhibitors selectively targeted the GA-dependent gene expression without turning on the ABA-dependent suppression of wheat embryo growth.

Taken together, these data suggested that the activation of TOR signaling takes place in the wheat scutellar epithelium and switches on critical steps of germination by inducing GAMYB and α-amylase synthesis.

### GA-Dependent Phosphorylation TaS6K Is Sensitive to Inhibitors of the TOR Kinase and GA Biosynthesis Inhibitor Paclobutrazol

A well-validated substrate of the mTOR kinase is a p70 ribosomal protein: S6K1. The phosphorylation state of this protein represents the TORC1 activity in animal and plant cells (Mahfouz et al., 2006; Meyuhas and Dreazen, 2009; Fenton and Gout, 2011; Xiong and Sheen, 2015). Using a tBLASTn search, we identified a *T. aestivum* cDNA encoding putative wheat S6K1 by searching for an AtS6K1 ortholog within the wheat genome. An alignment of the TaS6K1 protein sequence revealed a high similarity to human S6K1 (47% sequence identity, GenBank accession No. NP_001258989.1), AtS6K (63% sequence identity, GenBank accession No. NC_003074.8), and *Oryza sativa* S6K1 (89% sequence identity, GenBank accession No. ABF95793.1; Supplementary Figure S4).

**FIGURE 4:**
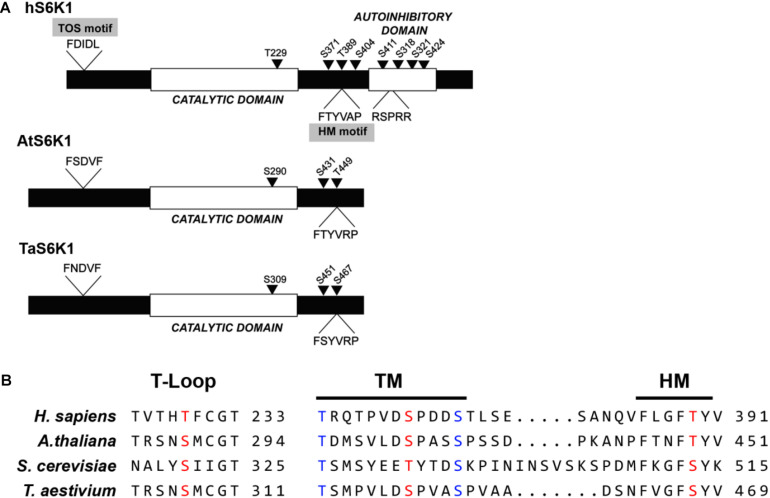
The protein kinase sequence analysis of *Triticum aestivum* ribosomal protein S6 kinase 1 (TaS6K1) and its alignment with the orthologs. **(A)** Comparison of human S6K1 (hS6K1), *A. thaliana* S6K1 (AtS6K1), and *T. aestivum* S6K1 (TaS6K1). hS6K1 contains a TOS-motif, catalytic domain, and autoinhibitory domain, whereas TaS6K1 and AtS6K1 have a putative catalytic domain. Possible phosphorylation sites in TaS6K1 were predicted based on the comparison with phosphorylation sites of hS6K1. Conserved and important serine (S) and threonine (T) residues are marked with arrows. **(B)** Sequence alignment of the T-loop, turn motif (TM), and hydrophobic motif (HM) of hS6K1, AtS6K1, TaS6K1, and *Saccharomyces cerevisiae* Ypk3.

The wheat S6K protein sequence consists of 481 amino acid residues with a predicted molecular mass of 53.6 kDa. N- and C-terminal sequences are least conserved between TaS6K1 and animal S6K1 proteins in contrast to their highly conserved kinase domains. Analysis of the kinase domain structure of TaS6K1 revealed an activation loop (T-loop) motif, turn motif (TM), and hydrophobic motif (HM) with their phosphorylation sites conserved in all S6 kinases belonging to the AGC kinase family (Figure 4). In *T. aestivum*, sites T-loop serine 309 (S309), TM S451, and HM serine 467 (S467) correspond to sites threonine 229 (T229), S371, and T389 in human S6K1 (Magnuson et al., 2012; Figures 4A,B). TaS6K1 resembles AtS6K and also lacks the TOR signaling (TOS) motif within its N terminus and the autoinhibitory loop at the C terminus.

Next, to determine the possible involvement of TaS6K1 in the GA signaling pathway, we prepared a cDNA encoding TaS6K1 from the wheat young leaves by RT-PCR. Sequencing results indicated that the CDS sequence of TaS6K1 is identical to the NCBI version of TaS6K1 (GenBank accession No. AK451448.1). To study the TaS6K1 protein, we developed a specific antibody. To this end, the 6xHis-tagged form of the rTaS6K1 protein was expressed in *E. coli* strain Rosetta (DE3) and purified by affinity chromatography. The purified rTaS6K1 protein was injected into rabbits to produce the antibody. Coomassie-stained gels revealed that the purified rTaS6K1 protein represented the main protein band and did not contain other contaminating proteins that migrated slightly below a 70-kDa protein marker (Supplementary Figure S1A). In an immunoblotting analysis, the affinity-purified polyclonal anti-TaS6K1 antibody detected a single protein band corresponding to a purified 6xHis-tagged rTaS6K1 protein (Supplementary Figure S1B) but not another 6xHis-tagged recombinant protein, *T. aestivum* Ape1L (TaApe1L) (Joldybayeva et al., 2014; Supplementary Figure S1B). The ELISA results indicated that the purified polyclonal antibody has high sensitivity to TaS6K1 (Supplementary Figure S5).

**FIGURE 5:**
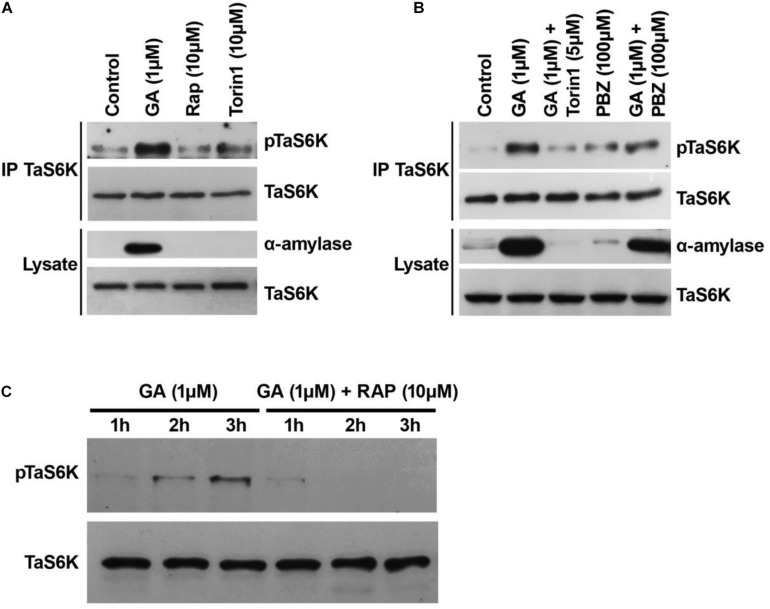
*Triticum aestivum* TOR (TaTOR) activity of isolated wheat embryos sensitive to TOR inhibitors and GA biosynthesis inhibitor paclobutrazol (PBZ). **(A)** The TaS6K1 phosphorylation is sensitive to the mTOR inhibitors. The wheat embryos dissected from 1-DAG seeds were incubated in 10 mM CaCl_2_ (control) with or without 1 μM GA or rapamycin or torin1 for 24 h. Rap, rapamycin. **(B)** The S467 phosphorylation level of TaS6K1 sensitive to the presence of paclobutrazol. The experiment was performed as in panel **(A)** using torin1 and PBZ as indicated. After 24 h, the embryos were lysed, TaS6K1 was immunoprecipitated, and the lysates and TaS6K1 immunoprecipitates were analyzed by immunoblotting for the indicated proteins or TaS6K phosphorylation. **(C)** Time course analyses of phosphorylation of TaS6K1 in wheat embryo cell-free extracts. Embryos were pre-treated with 100 μM PBZ for 20 h and were incubated with 1 μM GA and 10 mM CaCl_2_ in the absence or presence of 10 μM rapamycin for the indicated periods. After the incubation time was over, the embryos were lysed and analyzed for phosphorylation states of TaS6K1 by immunoblotting.

The site of TORC1-mediated phosphorylation in S6K1 is conserved between yeast and humans. In wheat, the hydrophobic-motif S467 site corresponds to the T389 site in the human S6K1 protein and to the T449 site in the *Arabidopsis* S6K1 protein (Figures 4A,B). The phosphorylation of S6K1 on T389 or T449 represents TORC1 activity in animal or *Arabidopsis* cells, respectively. Therefore, we analyzed the TORC1-dependent phosphorylation of TaS6K1 in wheat embryos after initial enrichment of the TaS6K1 protein by immunoprecipitation with the specific affinity-purified antibody. The immunopurified TaS6K1 protein from cell extracts of wheat embryos treated with GA alone, GA + rapamycin (10 μM), or GA + torin1 (10 μM) for 24 h was analyzed by immunoblotting with the phospho-specific S6K1 antibody recognizing the phosphorylated HM site on *A. thaliana* S6K1 (Cao et al., 2019). As shown in Figure 5A, TaS6K1 phosphorylation was clearly detectable in the cell extracts of wheat embryos exposed to GA. Rapamycin reduced the phosphorylation of TaS6K1 on S467 at the concentrations preventing the GA-induced α-amylase synthesis.

Our study suggests that the GA-dependent TOR activity initiates germination through induction of α-amylase expression in wheat embryos. To test whether the TOR activity is dependent on *de novo* GA biosynthesis, we incubated wheat embryos with 100 μM PBZ known as a potent inhibitor of GA biosynthesis (Takahashi et al., 1991). As shown in Figure 5B, the presence of 1 μM GA substantially increased the α-amylase level in the embryos when compared with control embryos. The inhibition of GA synthesis completely blocked the α-amylase production, suggesting that the inhibitor was effective in repressing the synthesis of gibberellins. The addition of GA reversed the PBZ effect by restoring the α-amylase production. The expression level of TaS6K1 in the GA-treated wheat embryos was similar to that in the control or PBZ-treated embryos (Figure 5B). High phosphorylation of TaS6K1 was detected in GA-treated wheat embryos but not in PBZ-treated embryos (Figure 5B).

The phospho-specific antibody recognizing the T389 site on S6K1 was also effective at monitoring the phosphorylation of TaS6K1 in cell-free extracts of the wheat embryos treated with GA (Figure 5C). In the presence of GA, the phosphorylation of TaS6K1 increased following 3 h of incubation. The addition of rapamycin along with GA resulted in a significant decrease in the TaS6K1 phosphorylation.

## Discussion

Our study reveals a role of TOR signaling in wheat embryo growth. This conclusion is based on our finding that mTOR inhibitors were effective in suppressing wheat germination.

The inhibitory impact of rapamycin on wheat germination in isolated embryos was more pronounced compared with its effect in whole seeds. The dose response implies that the rapamycin concentration was 10-fold higher in intact seeds than in isolated embryos. This finding suggests that the drug permeability is hindered substantially in intact seeds, and the isolated wheat embryo is more accessible to the treatment with rapamycin. Of note, in our study, a significant reduction in wheat seed growth was observed at 10 μM rapamycin. This concentration range is at least 100 times the concentration that inhibits yeast proliferation (Heitman et al., 1991) or that reduces the size and proliferation of mouse embryonic fibroblasts (Wicker et al., 1990; Sarbassov et al., 2006; Thoreen and Sabatini, 2009). It is likely that a high concentration of rapamycin is required because of poor penetration of rapamycin through the thick hemicellulosic wall of plant cells. Indeed, this notion is supported by the observation that rapamycin at 100 nM is effective at inhibiting an endogenous TOR protein kinase activity as revealed by phosphorylation of the T449 site of AtS6K1 and the T455 site of AtS6K2 in an isolated protoplast (Xiong and Sheen, 2012). Besides, rapamycin causes only weak growth inhibition of most higher plant species including *Arabidopsis*, *Nicotiana tabacum*, *Brassica napus*, cotton, and potato (Menand et al., 2002; Mahfouz et al., 2006; Sormani et al., 2007; Ren et al., 2012; Montane and Menand, 2013; Deng et al., 2016, 2017; Song et al., 2017). The insensitivity or low sensitivity to rapamycin can be attributed to a limited ability of plant FKBP12 proteins to form an inhibitory ternary complex with rapamycin owing to a lack of the critical amino acids mediating the interaction with rapamycin (Choi et al., 1996; Xu et al., 1998; Sormani et al., 2007). On the other hand, it has been reported that the growth of *Zea mays* and tomato [variety *Solanum lycopersicum* (SI)] is sensitive to rapamycin (Agredano-Moreno et al., 2007; Xiong et al., 2016). A functional study indicates that heterologous expression of tomato FKBP12 in *Arabidopsis* restores sensitivity to rapamycin (Xiong et al., 2016). It can be assumed that the rapamycin sensitivity in plants is species dependent, and more detailed studies are needed to determine why plants show different responses to the drug. Our study expands the list of rapamycin-sensitive plants by showing that rapamycin-dependent TaTOR signaling is launched during wheat seed germination.

Recent studies indicate that ATP-competitive inhibitors of the TOR kinase, including TORIN2, AZD-8055, WYE-132, and KU-63794, are more potent than rapamycin in inhibiting plant growth (Montane and Menand, 2013), and this phenomenon is linked to direct inhibition of the mTOR kinase activity. In our study, torin1 was ∼10-fold more effective than rapamycin at inhibiting the growth of the wheat seeds and of their isolated embryos. Indeed, torin1 exerted strong inhibition of root and shoot growth at 1 μM and caused a growth reduction in a concentration-dependent manner (Figures 2C,D). Altogether, the data indicate that rapamycin and torin1 most likely inhibit wheat seed growth by targeting the TaTOR protein.

The *Arabidopsis* genome contains one *TOR* gene (*AtTOR*) (Menand et al., 2002), two *RAPTOR* genes (Anderson et al., 2005; Deprost et al., 2005; Mahfouz et al., 2006; Rexin et al., 2015; Salem et al., 2018), and two *LST8* genes (Moreau et al., 2012). A loss-of-function mutation in *Raptor1b* leads to a significant delay in *Arabidopsis* seed germination (Salem et al., 2018). GA plays a crucial part in the regulation of early seed germination and breaking of dormancy (Weitbrecht et al., 2011). The breakdown of stored starch in the endosperm to support early seedling growth is an essential step for seed germination and subsequent seedling growth. At the early stage of germination, GA activates transcription factor GAMYB, which promotes *de novo* synthesis of α-amylases and an array of other hydrolases in the aleurone layer and embryo (Bethke et al., 1997). We hypothesized that the GA-dependent synthesis of α-amylase and consequent seed germination are mediated by the triggering of the TaTOR signaling pathway. We found that rapamycin specifically blocked GA-induced α-amylase gene expression in wheat embryos. In this experiment, the effective range of rapamycin was between 1 to 10 μM for inhibition, and complete inhibition was reached in a 10 μM range (Figure 3). Torin1 was more effective than rapamycin and inhibited α-amylase gene expression at 1 μM. The dose-dependent effects of rapamycin and torin1 on the GA-induced α-amylase gene expression are consistent with those seen in the germination experiment.

It is noteworthy that the TOR inhibitors selectively targeted the GA-dependent gene expression without affecting ABA-dependent *TaABI5* gene expression. The finding that the mTOR inhibitors suppressed GA-regulated transcription factor GAMYB (implicated in the induction of α-amylase genes) indicates that TOR signaling performs a function upstream of the GA-dependent transcription of *GAMYB* gene. These results point out that TOR signaling plays an important role in the GA-inducible expression of α-amylase.

A key substrate and mediator of the TOR protein kinase is S6K1, which is evolutionarily conserved between plants and humans (Fenton and Gout, 2011; Xiong and Sheen, 2012, 2014). To investigate the involvement of S6K1 (a key substrate and mediator of the TOR protein kinase) in GA signaling, we isolated the cDNA gene of TaS6K1, an uncharacterized substrate of TOR in wheat. Sequence homology indicates that *tas6k1* is the human *p70 s6k* gene ortholog, and its product is likely to be a direct target of the TOR kinase activity in wheat plants (Figure 4A,B). Western blotting with the antibody generated against TaS6K1 revealed that TaS6K1 was present in the control wheat embryos, and its expression was not induced by GA. By contrast, we detected increased TaS6K1 phosphorylation only in the presence of GA. Moreover, the TOR-dependent phosphorylation of a hydrophobic motif of TaS6K1 was sensitive to rapamycin and torin1 (Figure 5A).

The data presented here mean that GA switches on wheat germination processes by activating TaTOR signaling. Nonetheless, considering the antagonistic functions of ABA and GA, one could assume that the inhibition of the TOR kinase activity suppresses a GA response by decreasing the synthesis of ABA not by interfering with GA signaling.

Indeed, ABA, which induces polysome disassembly, inhibits cytokinin-induced phosphorylation of the 40S ribosomal S6 protein (Yakovleva and Kulaeva, 1987). More recently, it was demonstrated that the inhibition of TOR complex activity causes a significant decrease in ABA concentration and in the expression of genes *ZEP*, *NCED3*, and *AAO3* involved in ABA biosynthesis, in contrast to ABA-catabolic genes *CYP707A2* and *CYP707A3* (Kravchenko et al., 2015).

One way to examine the role of endogenous GA is to use an inhibitor of GA biosynthesis: PBZ. The latter inhibits GA biosynthesis by blocking cytochrome P450-dependent monooxygenases, thereby inhibiting oxidation of ent-kaurene into ent-kaurenoic acid (Rademacher, 2000).

In our study, gibberellin biosynthesis inhibitor PBZ blocked not only the α-amylase gene expression but also phosphorylation of TaS6K1 in wheat embryos. Exogenously applied GA reversed the PBZ effect by restoring the α-amylase production and phosphorylation of TaS6K1 (Figure 5B). Of note, in the presence of GA, the phosphorylation of TaS6K1 increased following 3 h of incubation (Figure 5C) and persisted over the course of 24 h (Figures 5A,B) in the PBZ-pre-treated embryos.

Thus, the data on gibberellin biosynthesis inhibitor support our theoretical model suggesting that GA regulates TOR–S6K1 signaling by launching the TaTOR kinase activity.

**FIGURE 6:**
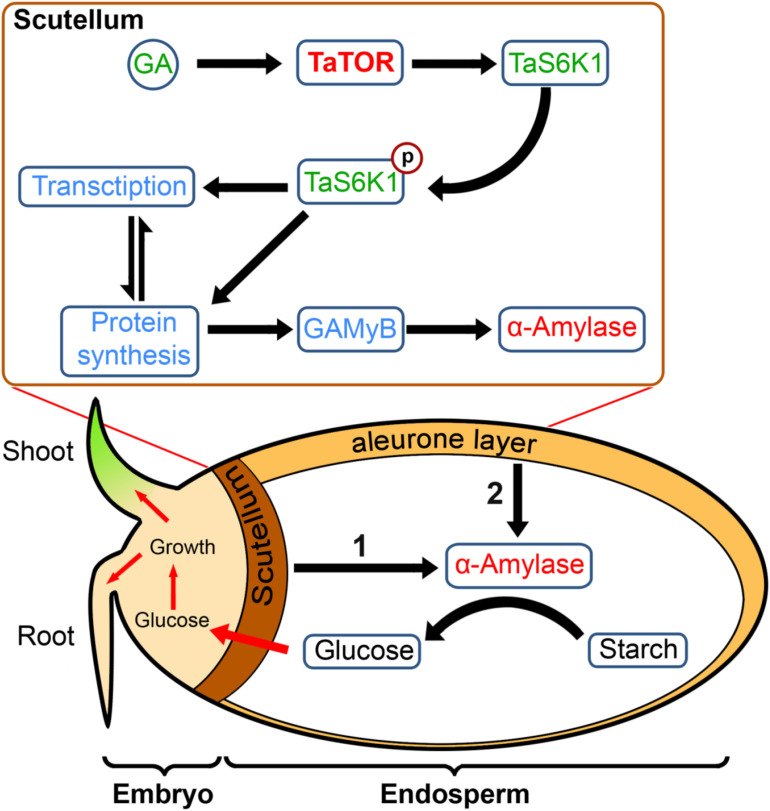
The model: a role of pTOR signaling in wheat germination. At the initial triggering stage of germination, GA by activating the TaTOR–TaS6K1 signaling induces the synthesis of GA-induced myb-like transcription factor (GAMYB) and α-amylase in wheat embryo scutella cells required for turning on the germination and seedling growth. An intensive α-amylase synthesis takes place later in aleurone cells following an initial stage of germination.

Dry seeds are heterotrophic; hence, germination depends on the energy accumulated in storage materials until the seedling reaches the autotrophic state. However, for starch in the endosperm to be converted to sugars, the embryonic scutellum and aleurone cell must synthesize secretory proteins such as α-amylase (Figure 6). In cereals, the α-amylase synthesis first initiated in the scutellum of embryos and followed by the aleurone layer (Subbarao et al., 1998). Synthesis of α-amylase requires a large pool of available amino acids, which is likely to come from the protein storage vacuoles (Bethke et al., 1997). From our results, we propose that, in wheat embryo scutella cells, the effect of GA directly or indirectly can mediate TOR activation, triggering phosphorylation of TaS6K1 at the TOR-specific hydrophobic motif residue S467, which promotes the induction of synthesis of α-amylase and therefore the initiation of germination and subsequent seedling growth (Figure 6).

How GA can activate TaTOR signaling is an attractive puzzle to pursue. Activation of mTORC1 in animal cells by amino acids depends on the small GTPase RagA/B or RagC/D or small GTPase Rheb by growth factor signaling (Shen et al., 2017). In plant *A. thaliana*, auxin can activate TOR via Rho-like small GTPase 2 (ROP2) (Schepetilnikov et al., 2017). Of note, two putative Rho GTPase genes were found to be upregulated 17- to 40-fold in response to GA in barley (Chen and An, 2006). It will be interesting to determine whether high expression of GA-dependent Rho GTPases is sufficient to trigger TaTOR kinase activity. Most likely, a biochemical characterization of the TaTOR complex will be a most informative study in identifying a GA-dependent regulatory component of Ta-TOR complex that has been critical in identifying a role of the Rag GTPases in a nutrient-dependent regulation of mTORC1.

## Conclusion

In conclusion, the presented data indicate that GA-dependent activation of TOR–S6K1 signaling turns on active synthesis of α-amylase required for wheat embryo growth.

## Data Availability Statement

The raw data supporting the conclusions of this article will be made available by the authors, without undue reservation.

## Ethics Statement

The animal study protocol was reviewed and approved by the Ethics Committee on the Bioethics of the Scientific Center for Anti-Infectious Drugs (SCAID), Almaty, Kazakhstan.

## Author Contributions

BS, SA, IS, and AM conducted the experiments. AB, DS, and MS wrote the manuscript and analyzed the data. All authors contributed to the critical review of the manuscript and approved the submitted version.

## Conflict of Interest

The authors declare that the research was conducted in the absence of any commercial or financial relationships that could be construed as a potential conflict of interest.

## Supplementary Material

Supplementary Figure 1 Detection of the purified TaS6K1 protein by the anti-TaS6K1 antibody. **(A)** SDS-PAGE analysis of the purified recombinant TaS6K1 and TaApe1L proteins. Lane 1, protein size markers; lane 2, TaS6K1, 0.5 μg; lane 3, TaApe1L, 1 μg; Western blot analysis with anti-TaS6K1 **(B)**, anti-RPS6KB1 (Ab-389) **(C)**, anti-TaApe1L **(D)**, and anti-His antibodies **(E)**. In all four western blots, 1 μg of purified 6xHis-APE1L was loaded in the first lane and 0.5 μg of purified 6xHis-TaS6K1 in the second lane.

Supplementary Figure 2 The effect of rapamycin and ATP-competitive TOR inhibitors on isolated wheat embryos. The embryos were incubated on the 0.5× MS medium supplemented with ABA alone and rapamycin, torin1, or pp242 as indicated for 4 day incubation.

Supplementary Figure 3 The influence of TOR kinase inhibitors on mRNA levels of the indicated genes *GAMYB*, α-amylase, and *ABI5* in wheat embryos. The wheat embryos dissected from 1-DAG seeds were incubated for 24 h in the presence 10 mM CaCl_2_ (control) with or without 10 μM ABA, or 1 μM GA, or 1 μM GA with rapamycin or torin1 as indicated. *TaABI5*, *GAMYB*, and α-amylase cDNAs were amplified by RT-PCR with gene-specific primers. Ribosomal RNA was included as an internal control of sample loading. Rap: rapamycin.

Supplementary Figure 4 Protein sequence alignment of putative *T. aestivum* S6K1, *A. thaliana* S6K1, *Oryza sativa* S6K1, and human S6K1. The deduced amino acid sequences were aligned in the ClustalX 2.1 software. Asterisks (^∗^), colons (:), and periods (.) indicate identical, conserved, and semiconserved aligned residues, respectively.

Supplementary Figure 5 The titer of antiserum according to the ELISA. The purified antibody was subjected to serial dilution (from 1000- to 128,000-fold) and reacted with the purified rTaS6K1 protein. Preimmunization rabbit serum served as a negative control. The antibody titer is defined as the highest dilution of serum at which the A_405_ ratio (A_405_ of post-immunization serum/A_405_ of preimmunization serum) is greater than 2:1. Data are presented as the mean ± standard deviation.

## Abbreviations

ABA: abscisic acid
FKBP12: FK506-binding protein 12 kDa
GA: gibberellic acid
GAMYB: GA-induced myb-like transcription factor
mTOR: mammalian TOR
PBZ: paclobutrazol
TaABI5: *Triticum aestivum* ABA response element-binding factor
TaS6K1: *Triticum aestivum* ribosomal protein S6 kinase 1
TOR: target of rapamycin.
