# Supplementary material for: Wheat Germination Is Dependent on Plant Target of Rapamycin Signaling
Source: Front Cell Dev Biol. 2020 Nov 23;8:606685. doi: 10.3389/fcell.2020.606685 (PMC7719826; doi:10.3389/fcell.2020.606685)
Supplement: Supplementary Figure 1 — Detection of the purified TaS6K1 protein by the anti-TaS6K1 antibody. (A) SDS-PAGE analysis of the purified recombinant TaS6K1 and TaApe1L proteins. Lane 1, protein size markers; lane 2, TaS6K1, 0.5 μg; lane 3, TaApe1L, 1 μg; Western blot analysis with anti-TaS6K1 (B), anti-RPS6KB1 (Ab-389) (C), anti-TaApe1L (D), and anti-His antibodies (E). In all four western blots, 1 μg of purified 6xHis-APE1L was loaded in the first lane and 0.5 μg of purified 6xHis-TaS6K1 in the second lane. [file Data_Sheet_1.pdf]

**Supplementary Information**

# Wheat Germination Is Dependent on Plant Target of Rapamycin Signaling

**Bauyrzhan Smailov[1,2], Sanzhar Alybayev[1,2], Izat Smekenov[1,2], Aibek Mursalimov[1,2], Murat Saparbaev[1,3,*], Dos Sarbassov[4,*] and Amangeldy Bissenbaev[1,2,*]**

[1]Department of Molecular Biology and Genetics, Faculty of Biology and Biotechnology, al-Farabi Kazakh National University, Almaty, Kazakhstan
[2]Scientific Research Institute of Biology and Biotechnology Problems, al-Farabi Kazakh National University, Almaty, Kazakhstan
[3]Groupe «Mechanisms of DNA Repair and Carcinogenesis», Equipe Labellisée LIGUE 2016, CNRS UMR9019, Université Paris-Sud, Gustave Roussy Cancer Campus, F-94805 Villejuif Cedex, France
[4]Department of Biology, Nazarbayev University, Nur-Sultan, Kazakhstan

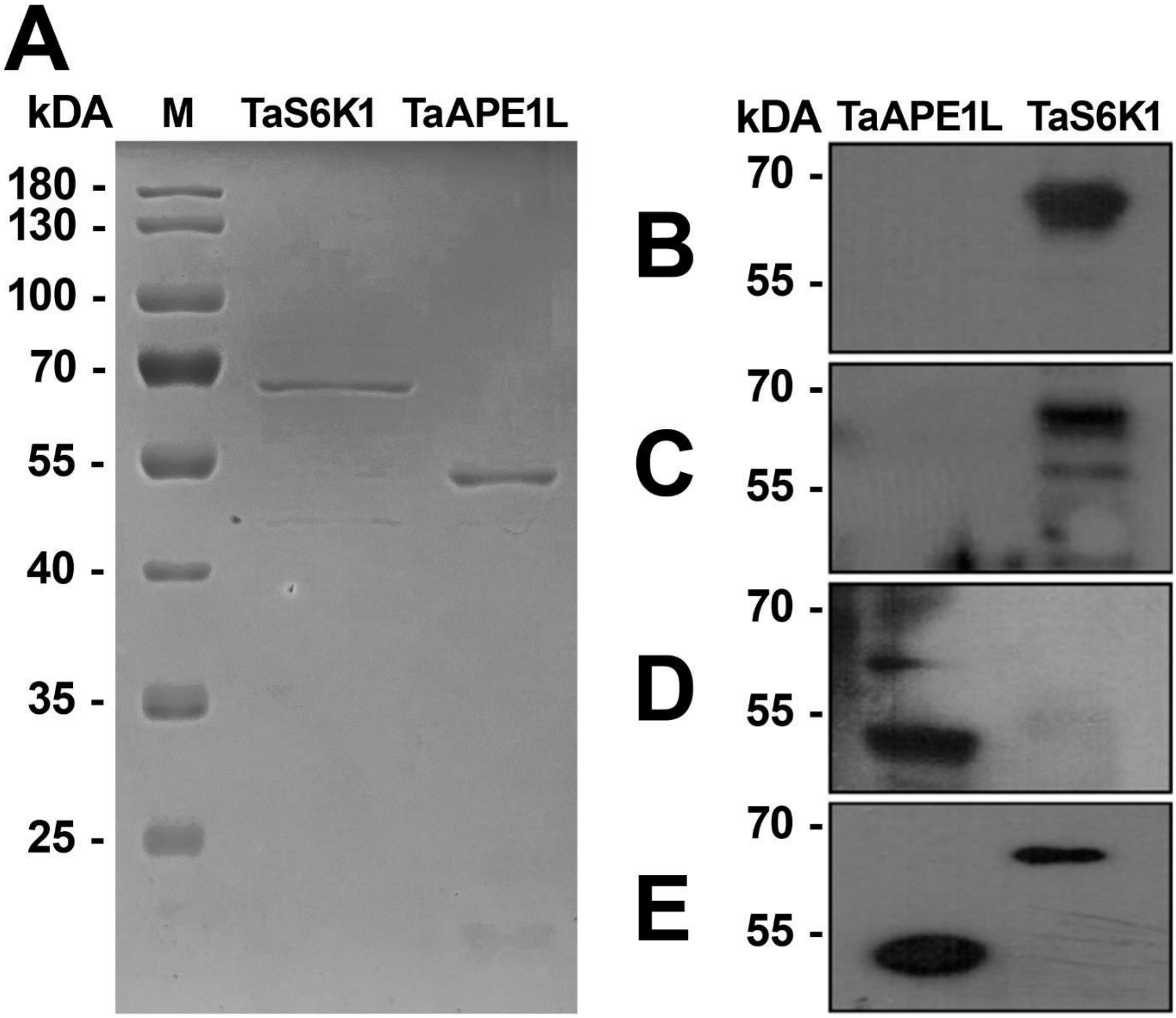


**Supplementary Figure S1.** Detection of the purified TaS6K1 protein by the anti-TaS6K1 antibody. (A) SDS-PAGE analysis of the purified recombinant TaS6K1 and TaApe1L proteins. Lane 1, protein size markers; lane 2, TaS6K1, 0.5 μg; lane 3, TaApe1L, 1 μg; Western blot analysis with anti-TaS6K1 (B), anti-RPS6KB1 (Ab-389) (C), anti-TaApe1L (D), and anti-His antibodies (E). In all four western blots, 1 μg of purified 6xHis-APE1L was loaded in the first lane and 0.5 μg of purified 6xHis-TaS6K1 in the second lane.

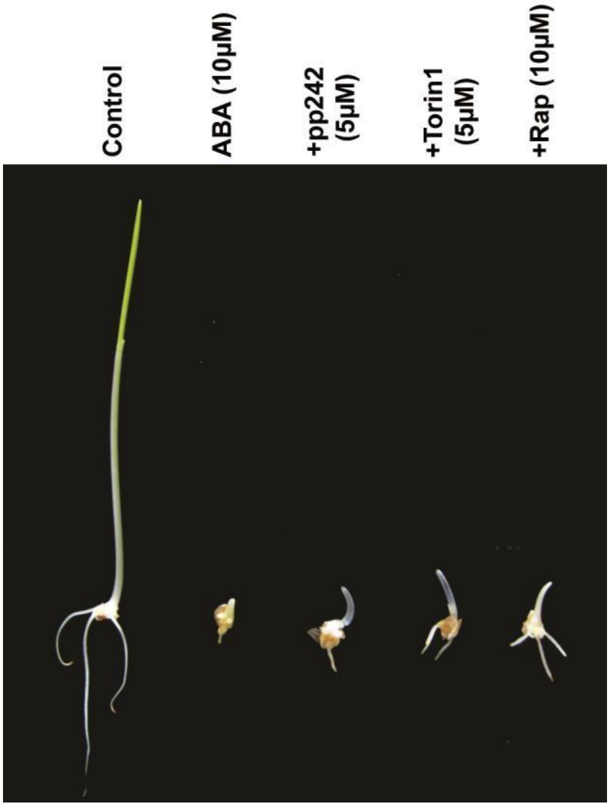


**Supplementary Figure S2.** The effect of rapamycin and ATP-competitive TOR inhibitors on isolated wheat embryos. The embryos were incubated on the 0.5× MS medium supplemented with ABA alone and rapamycin, torin1, or pp242 as indicated for 4-day incubation.

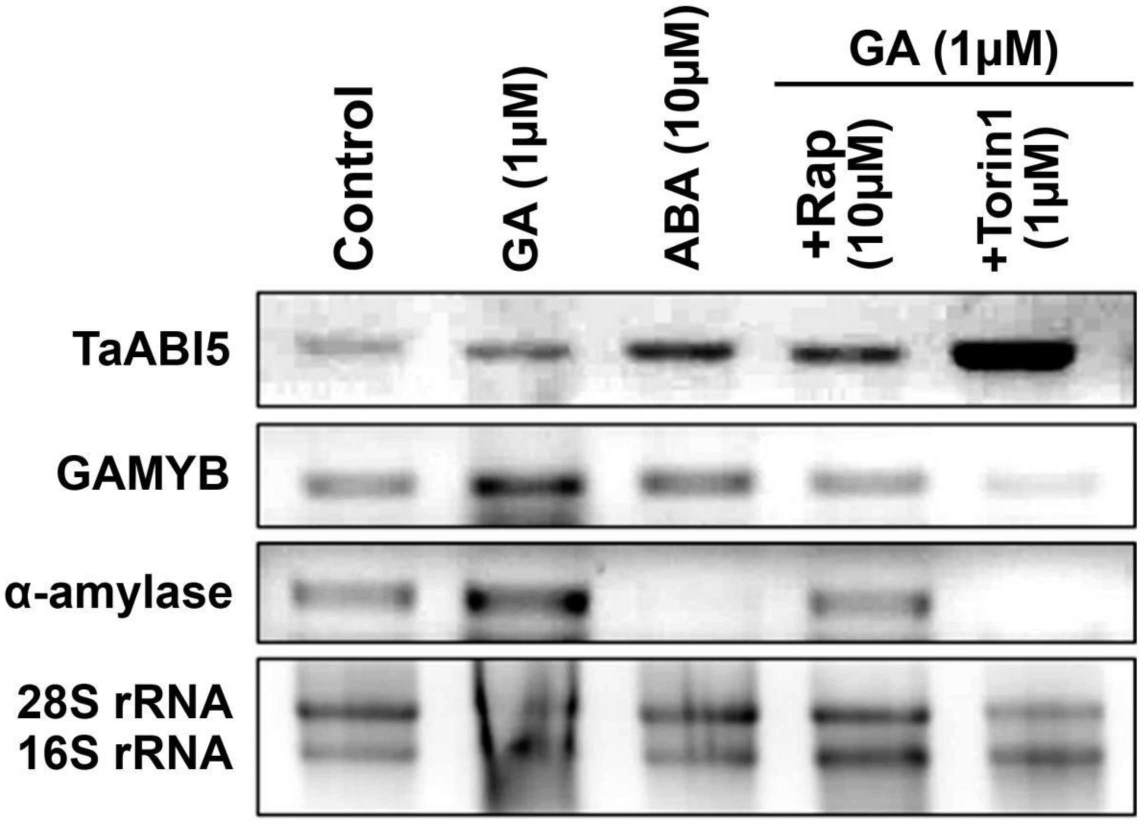


**Supplementary Figure S3**. The influence of TOR kinase inhibitors on mRNA levels of the indicated genes GAMYB, α-amylase, and ABI5 in wheat embryos. The wheat embryos dissected from 1-DAG seeds were incubated for 24 h in the presence 10 mM $CaCl_2$ (control) with or without 10 μM ABA, or 1 μM GA, or 1 μM GA with rapamycin or torin1 as indicated. TaABI5, GAMYB, and α-amylase cDNAs were amplified by RT-PCR with gene-specific primers. Ribosomal RNA was included as an internal control of sample loading. Rap: rapamycin.

```
p70S6K ------------------------------------------MAGVFDI---------DL 9
AtS6K1 MVSS---------QRPVPNKIQKQQYLSISPSNSVLKDDVELEFSDVFGPLPE-------- 44
OsS6K1 MVSSEISSVTTTHAQGPKLFRGKILLPMGPPDVVPSENVEFDFSDVFGPTAVQTPTDLSI 60
TaS6K1 MVSSEIPSVTTTHTQRPKLFTGMILLPKGPPDVVLPENVEFDFNDVFGATAVQTPTEVSI 60
                                                 : .**.

p70S6K DQPEDA----GSEDELEEGGQLNESMDHGGVGPYELG-------------------MEHC 46
AtS6K1 ----------EANDIAYDEPAVVYSRSHSLVGPCSLDSHSLKLTKLTLLETEDSIDLVEC 94
OsS6K1 LTPDSPAPLTESSEGIYNDPLVIVKRSHSLVGPSSLVSQSLPLSKLTLHESDSALDLLEC 120
TaS6K1 LTPGSPAPLAESNEEVYNDPVVITKRSHSLVGPTSLVSQSLPLSKLTLHESESSLDLLEC 120
                  :.:   :   :  . .*. *** .*                    : .*

p70S6K EK------------------FEISETSVNRGPEKIRPECFELLRVLGKGGYGKVFQVRKV 88
AtS6K1 LEGESLKENDDFSGNDDSDNEKALEGDLVKVSGVVGIDDFEVMKVVGKGAFGKVYQVRKK 154
OsS6K1 TK-EKKSNQEALSDEEL--------DDTKNENGVVGLDDFEVLKLVGQGAFGKVFQVRKK 171
TaS6K1 LSKEKKSGQGSLSDEEL--------NDTTKENEAVGLDDFELLKLVGQGAFGKVYQVRRK 172
        .                        .  .    :  : **:::::*:*.:***:***:

p70S6K TGANTGKIFAMKVLKKAMIVRNAKDTAHTKAERNILEEVKHPFIVDLIYAFQTGGKLYLI 148
AtS6K1 ---ETSEIYAMKVMRKDHIMEKN-HAEYMKAERDILTKIDHPFIVQLKYSFQTKYRLYLV 210
OsS6K1 ---GTSEIYAMKVMRKDKILEKN-HAEYMKAERDILTKVDHPFVVQLRYSFQTKYRLYLV 227
TaS6K1 ---CTSDIYAMKVMRKDKILEKN-HAEYMKAERDILTKVDHPFVVQLRYSFQTKYRLYLV 228
           *..*:****::*  *:.:  .: : ****:** ::.***:*:* *:***  :***:

p70S6K LEYLSGGELFMQLEREGIFMEDTACFYLAEISMALGHLHQKGIIYRDLKPENIMLNHQGH 208
AtS6K1 LDFINGGHLFFQLYHQGLFREDLARVYTAEIVSAVSHLHEKGIMHRDLKPENILMDTDGH 270
OsS6K1 LDFINGGHLFFQLYQQGLFREELARIYTAEIVSAVAHLHANGIMHRDLKPENILLDADGH 287
TaS6K1 LDFVNGGHLFFQLYQQGLFREELARIYTAEIVSAVAHLHANGIMHRDLKPENILLDAHGH 288
       *:::.**.**:** ::*:* *: * .* ***  *:.*** :**::********::  .**

p70S6K VKLTDFGLCKESIHDGTVTHTFCGTIEYMAPEILMRSGHNRAVDWWSLGALMYDMLTGAP 268
AtS6K1 VMLTDFGLAKEF-EENTRSNSMCGTTEYMAPEIVRGKGHDKAADWWSVGILLYEMLTGKP 329
OsS6K1 AMLTDFGLAKEF-DENTRSNSMCGTVEYMAPEIVQGRGHDKAADWWSVGILLFEMLTGKP 346
TaS6K1 AMLTDFGLAKEF-DENTRSNSMCGTVEYMAPEIVQGRGHDKAADWWSVGILLFEMLTGKP 347
       . ******.**  .:.* ::::*** *******:   **::*.****:* *:::**** *

p70S6K PFTGENRKKTIDKILKCKLNLPPYLTQEARDLLKKLLKRNAASRLGAGPGDAGEVQAHPF 328
AtS6K1 PFLGS-KGKIQQKIVKDKIKLPQFLSNEAHAILKGLLQKEPERRLGSGLSGAEEIKQHKW 388
OsS6K1 PFVGGNRDKVQQKIVKEKIKLPAYLSSEVHSLLKGLLHKEAGRRLGCGPGGSNEIKNHKW 406
TaS6K1 PFFGGNRDKIQQKIVKEKMKLPTYLSSEVHSLLKGLLHKEAGRRLGSGPGGSDEIKNHKW 407
       ** *  : *  :**:* *::** :*:.*.: :** **:::   ***.* ..: *:: * :

p70S6K FRHINWEELLARKVEPPFKPLLQSEEDVSQFDSKFTRQTPVDSPDDSTLSESANQVFLGF 388
AtS6K1 FKGINWKKLEAREVMPSFKPEVSGRQCIANFDKCWTDMSVLDSPASSPSSDPKANPFTNF 448
OsS6K1 FKSVNWKKLDSRQIQPSFRPNVAGKTCIANFDECWTSMPVLDSPVASPVAA--DSNFVGF 464
TaS6K1 FKAVNWKKLEARQITPSFCPNVAGQTCIANFDECWTSMPVLDSPVASPVAA--DSNFVGF 465
       *: :**::* :*:: * * * : ..  :::**. :*    :***  *  :    . * .*

p70S6K TYVAPSVLESVKEKFSFEPKIRSPRRFIGSPRTPVSPVKFSPGDFWGRGASASTANPQTP 448
AtS6K1 TYVRPPPSFLHQSTTTL*------------------------------------------ 465
OsS6K1 SYVRPAPFLQRPSPLG*------------------------------------------- 480
TaS6K1 SYVRPEPFLQKPSPLG*------------------------------------------- 481
       :** *       .

p70S6K VEYPMETSGIEQMDVTMSGEASAPLPIRQPNSGPYKKQAFPMISKRPEHLRMNL* 502
AtS6K1 ------------------------------------------------------- 465
OsS6K1 ------------------------------------------------------- 480
TaS6K1 ------------------------------------------------------- 481
```

**Supplementary Figure S4**. Protein sequence alignment of putative *T. aestivum* S6K1, *A. thaliana* S6K1, *Oryza sativa* S6K1, and human S6K1. The deduced amino acid sequences were aligned in the ClustalX 2.1 software. Asterisks (*), colons (:), and periods (.) indicate identical, conserved, and semiconserved aligned residues, respectively.

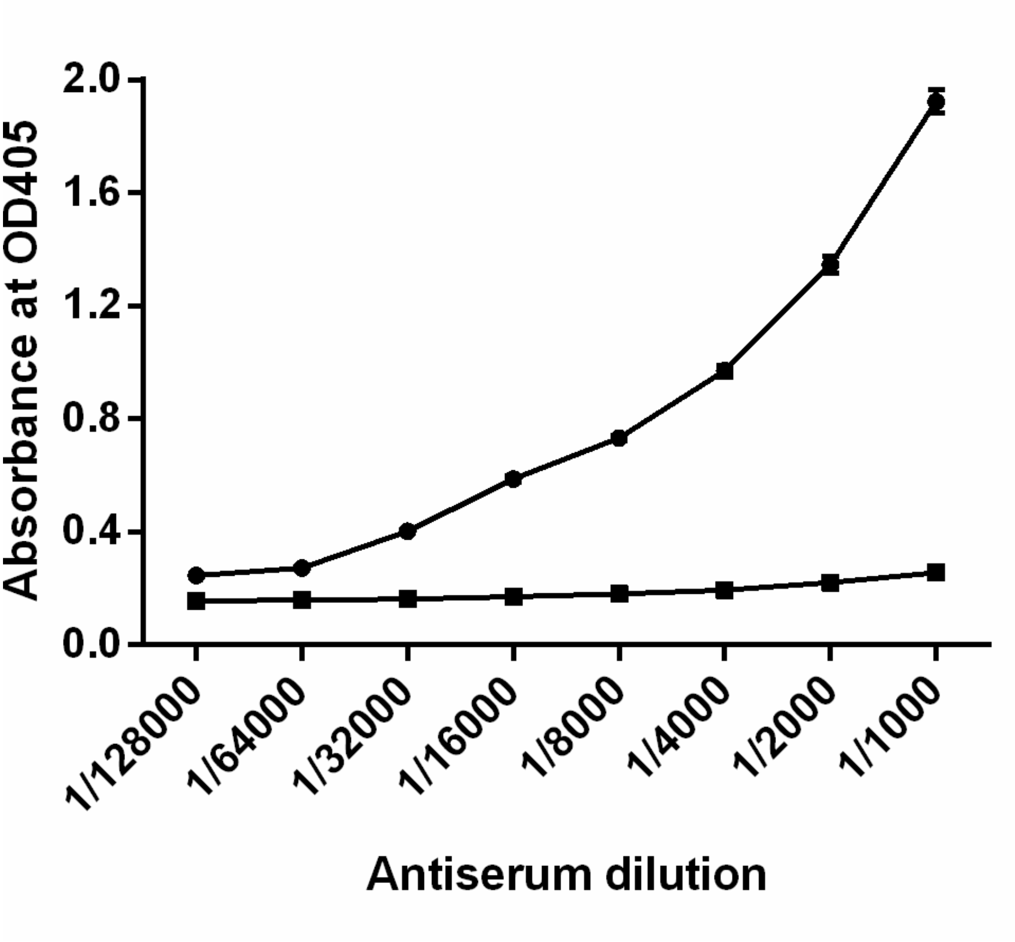


**Supplementary Figure S5**. The titer of antiserum according to the ELISA. The purified antibody was subjected to serial dilution (from 1000- to 128,000-fold) and reacted with the purified rTaS6K1 protein. Preimmunization rabbit serum served as a negative control. The antibody titer is defined as the highest dilution of serum at which the $A_{405}$ ratio ($A_{405}$ of postimmunization serum/$A_{405}$ of preimmunization serum) is greater than 2:1. Data are presented as the mean ± standard deviation.
